# 自体肺叶再植治疗上叶中心型肺癌

**DOI:** 10.3779/j.issn.1009-3419.2010.11.09

**Published:** 2010-11-20

**Authors:** 玉伦 杨, 文增 赵

**Affiliations:** 4500052 郑州，郑州大学第一附属医院心胸外科 Department of Thoracocardiac Surgery, the First Affiliated Hospital of Zhengzhou University, Zhengzhou 450052, China

**Keywords:** 肺肿瘤, 肺切除术, 自体肺再植, Lung neoplasms, Pneumonectomy, Lung autotransplantation

## Abstract

**背景与目的:**

肺癌患者行根治性全肺切除术后生活质量往往较差，最大限度地保留健康肺组织具有临床价值。本文报道3例自体肺再植在上叶中心型肺癌治疗中的应用和经验总结。

**方法:**

本组共3例患者，肿瘤侵犯支气管或转移性淋巴结融合包绕、累及主肺动脉，肿瘤跨越斜裂侵及下叶边缘，先做完全性全肺切除术，体外低钾右旋糖酐液（low-potassium dextran, LPD）顺、逆行灌注后从离体标本中摘取可保留的下肺叶；下肺静脉再植于上肺静脉残端，按肺静脉、支气管、肺动脉顺序依次吻合。

**结果:**

手术总时间220 min-250 min，下叶肺离体时间120 min-150 min；术后3 d-5 d拔除胸管，胸部X线示再植肺膨胀良好；术后随访4个月-8个月，完成3个或4个周期辅助化疗，患者生活质量良好。

**结论:**

自体肺叶再植是一种可行的、可供选择的肺癌完全切除术式，是最大限度地切除病变和保留健康肺组织的理想术式。

## Abstract (en)

**Background and objective:**

Patients suffering from lung cancer often have poor quality of life after pneumonectomy. It has clinical significances to preserve maximum lobes of the "healthy" lung. The aim of this study is to report the applications of lung replantation in treatment of superior lobe central lung cancer.

**Methods:**

Three lung cancer cases were included and analysed. The bronchus and margin of lower lung lobe were encroached by cancer. Pulmonary artery was invaded and surrounded by metastatic lymph node. Complete pneumonectomy, antegrade perfusion and retroperfusion with low-potassium dextran (LPD) solution *in vitro* were performed. The retainable lower pulmonary lobe was selected from the isolated lung and superior pulmonary vein was replaced with inferior pulmonary veins. The bronchus and pulmonary artery were inosculated by turns.

**Results:**

The operative cumulative time ranged from 220 min to 250 min. The isolated time of lobus inferior pulmonary ranged from 120 min to 150 min. The chest tube was pulled out after chest X-ray confirmed the reimplant lung full re-expansion. The patients were followed up for 4 months to 8 months and accomplished adjuvant chemotherapy for 3 or 4 periodicities. The patients had a sound quality of life.

**Conclusion:**

Lung replantation removing the extensive tumor tissue and retaining the maximum pulmonary normal tissue is an useful method for treatment of lung cancer.

1985年Toomes等^[1]^在1例双袖状右上、中肺叶联合切除时，因肺动脉切除过长而将下肺静脉再植于上肺静脉残端完成了肺动脉吻合。于是这种自体肺再植术使手术治疗Ⅲ期中心型肺癌向前迈进了一步，是一种尽可能保全患者肺组织的肺癌根治术式，以往被用于心肺功能不能耐受全肺切除，而肿瘤累及肺动脉或主支气管长度过长，因下肺静脉的牵扯不能完成双袖状肺叶切除术的Ⅲ期上叶中心型肺癌。为尽可能地提高患者术后生存质量，在可以满足全肺根治性切除的条件下我们进行了3例自体肺叶再植，术后患者恢复良好。现就手术方法和经验体会报告如下。

## 1 临床资料

例1，男，年龄50岁，右中心型肺癌。因“间断咳嗽、痰血、胸闷2个月”入院，伴右侧胸痛及发热（39 ℃）。胸部增强CT检查提示右上肺纵隔旁见不规则软组织团块影，病灶与纵隔分界不清，右肺上叶不张，肿瘤及肿大淋巴结融合成团包绕右肺动脉主干致管腔明显狭窄变形。电子支气管镜检查：自右主支气管前壁（距隆凸嵴1个软骨环）至右上叶支气管口、右上叶分嵴及右中间干粘膜浸润、肥厚，右上叶支气管口闭塞；右中间干下段前壁见0.8 cm×1.0 cm新生物，右中下肺叶远端及左侧各级支气管形态、粘膜大致正常。病例报告为中、低分化鳞癌。头颅CT及全身骨扫描无异常。肺功能FEV_1_ 2.55 L，占预计值78%，血气分析显示PO_2_ 101.9 mmHg。

全身麻醉双腔气管插管右第5肋间前外侧切口开胸探查，肺裂发育良好，右肺上叶呈实变性不张，近肺门不规则质硬肿块约4 cm×5 cm，肺门呈冻结状，3、4组淋巴结增大明显融合成团，中、下肺叶无明显肿瘤侵及。切开心包充分暴露肺动脉圆锥、右肺动脉干心包内段和上（上、中叶肺静脉心包内共干）、下肺静脉，见肿瘤侵犯心包内段肺动脉，距离肺动脉圆锥近2.0 cm，上、下肺静脉脉未见肿瘤侵及。打开水平裂见肿瘤包绕上叶肺动脉前干及后升支，中叶肺动脉内侧支距肿瘤约0.5 cm，解剖出基底干肺动脉。打开纵隔胸膜清除3、4及7组淋巴结，探查右主支气管及隆凸后判定可行右全肺心包内根治性切除术。结合术前电子支气管镜检查结果，需切除整个右主及中间干支气管，肺动脉及支气管切除长度均较长。首先进行全身半肝素化，心耳钳距肺动脉圆锥1.0 cm处阻断右肺动脉血流，后血管闭合器切断上、下肺静脉，这样大大缩短了下叶肺离体时间，注意多保留下肺静脉长度，最后距隆凸嵴0.5 cm切断右主支气管。将右肺置于4 ℃肝素溶液中（12 500 U/500 mL生理盐水），首先自主肺动脉顺行灌注4 ℃低钾右旋糖酐液（low-potassium dextran, LPD）（LPD液1 000 mL+PGE 1 250 μg+NaHCO_3_ 10 mL），肺变苍白后改下肺静脉逆行灌注，灌注压约为28 cm H_2_O-30 cm H_2_O。按根治性切除清扫区域淋巴结并严密止血，后平中叶支气管开口切断中间干见肿瘤性结节，牺牲右肺中叶并尽可能地保留下叶支气管长度。将离体下肺置入胸腔，先进行下叶肺静脉与上肺静脉残端的5-0 Prolene连续外翻缝合，开放阻断钳左心房血液逆流涌入肺静脉，自基底干肺动脉流出无血凝块的血液后无损伤血管钳夹闭；将下叶支气管残端修成斜面，3-0可吸收微桥缝线行膜部连续、软骨部单纯间断缝合；最后5-0 Prolene连续外翻将右下叶基底干肺动脉与左肺动脉干吻合。恢复通气及血液循环，查无活动性出血及水试吻合口无漏气，用化学胶喷洒支气管吻合口。术后病理报告：右上肺中心型鳞癌（直径4.5 cm），支气管切缘未见癌细胞，纵隔淋巴结（3、4、10组）见癌转移。

下叶肺离体时间15 min，肺动脉总阻断时间140 min，手术总时间245 min。术后第1天患者即自行咳痰，术后第2天常规给予床边纤维支气管镜吸除血痰，发现吻合口通畅、光整；胸片示肺膨胀良好，拔除胸管。术后给予“多西他赛+卡铂”4个周期辅助化疗，随访6个月生活治疗较好，恢复轻体力劳动。

例2，男，48岁，左上肺神经内分泌癌（不典型类癌）。因“间断咳嗽、痰血2年余”入院，伴发热、乏力及盗汗，按“肺结核”抗痨治疗1年，胸部CT提示左肺门团块影并左上肺不张，纵隔淋巴结增大呈团包绕左肺动脉干；电子支气管镜报告自左上叶支气管长出的类圆形新生物遮盖了左上叶分嵴，左下叶支气管口粘膜略充血、粗糙，其所属各级及右侧支气管形态、粘膜大致正常，活检提示不典型类癌。肺功能FEV_1_ 2.98 L，占预计值94.3%，血气分析显示PO_2_ 89.6 mmHg。

左第5肋间前外侧切口开胸探查，肺裂发育欠佳，舌叶大部与下肺叶不发育，第5组及肺门淋巴结明显肿大融合呈冻结状，打开纵隔胸膜及心包，见肿瘤包绕左肺动脉干心包内段，距肺动脉圆锥仅1 cm，左上、下肺静脉未见肿瘤侵犯；解剖基底干肺动脉亦未见肿瘤侵及，在肺动脉圆锥的左侧壁上心耳钳阻断左肺动脉血流，距肺动脉圆锥0.5 cm处切断左肺动脉干，心包内血管闭合器切断左上、下肺静脉。在距隆突1.5 cm处切断左主支气管，左肺上叶、下叶肺离体。灌注完成后肺组织直线切缝器切除发育不全之肺裂，检查下肺叶支气管粘膜基本正常。将离体的下肺叶重置胸腔内，依次吻合静脉、支气管、肺动脉，下叶肺离体时间12 min，肺动脉总阻断时间125 min，手术总时间225 min。术后第2天床边纤维支气管镜检查发现吻合口主支气管侧一处活动性点状渗血，给予内镜下烧灼止血，第4天拔除胸管。术后3周应用“重组人血管内皮抑制素+顺铂”联合化疗3次，随访4个月生活基本自理。

例3，女，60岁，左肺上叶小细胞癌。因“间断咳嗽、咳痰伴胸闷20 d”入院。胸部CT报告左肺上叶占位性病变，左肺上叶不张，主动脉窗淋巴结肿大明显，包绕压迫左肺动脉干起始部；电子支气管镜提示左主支气管下段粘膜充血、增厚，左肺上叶支气管口见新生物完全堵塞，左肺下叶所属各段管口均牵拉扭曲变形；右侧各叶段管口通畅，粘膜光滑，活检病例报告见“癌细胞”。肺功能FEV_1_ 2.29 L，占预计值108.5%，血气分析显示PO_2_ 77 mmHg。开胸探查见上叶近肺门处肿块5 cm×5 cm×4.5 cm，下极靠近斜裂并与下叶内前基底段粘连较重，但下肺叶内未触及可疑结节；肺门见多个肿大淋巴结，打开心包暴露左肺动脉主干及上叶肺静脉，肿块与二者粘连严重，距离肺动脉圆锥约1.2 cm，上叶肺静脉心包内尚有1.0 cm处理长度。行左上肺双袖状切除，含主支气管、肺动脉干各4 cm，由于下肺静脉牵拉，肺动脉两断端并拢张力大，血管闭合器切断下肺静脉，离体肺灌注后以肺直线切缝器切除受影响部分内基底段，类似手术方法完成3处吻合。下叶肺离体时间14 min，肺动脉总阻断时间132 min，手术总时间230 min。术后2 d胸片提示左余肺不张，呈高密度影，纤维支气管镜吸出大量陈旧性血痰后给予左胸腔持续负压吸引，复查胸片肺复张良好，第5天拔除胸管。术后4周应用“环磷酰胺+阿霉素+顺铂”化疗4次，随访8个月生活状况良好，可从事一般家务劳动。

## 2 讨论

Ⅲ期肺癌指无远处转移、局限于胸腔内的中晚期肺癌，常常侵犯肺动脉干、主支气管和隆突等，因手术难度高、危险性大，往往被迫放弃手术行内科治疗或被迫行心包内全肺切除术，疗效极差且术后部分患者往往肺功能欠佳，生活质量低下。近年来支气管袖式肺叶、支气管肺动脉袖式肺叶切除已经成为治疗Ⅲ期中心型肺癌的常规术式，全肺切除术在肺癌外科治疗中所占的比例逐年下降。然而，当肿瘤需要切除的范围较大或肿瘤侵犯主支气管或肺动脉干、切除的肺动脉或支气管较长时，由于肺静脉的牵拉，环形切开肺下静脉周围心包后支气管或肺动脉的两断端仍不能并拢而无法完成吻合，此时把下肺静脉再植于上肺静脉残端的自体肺再植术，成为最大限度地切除病变和保留健康肺组织的理想术式。

### 2.1 充分的围手术期准备及管理

术前患者一定要行完善的术前检查。包括肺部CT检查明确肿瘤的浸润范围和肿瘤与支气管的关系；行纤维支气管镜检查明确支气管内肿瘤侵犯程度，尤其是肿瘤是否浸润主支气管及其距隆突的长度；头颅CT及全身骨ECT检查有无远处转移；肺功能学检查明确患者肺功能情况；肝肾功能及心功能检查明确患者一般情况。给予布地奈德雾化吸入及应用解痉祛痰药物，加强营养支持，进行呼吸功能锻炼，练习深咳和腹式呼吸。

### 2.2 全面细致的术中探查

术中对主肺动脉干、总支气管和肺静脉进行全面详细的探查。首先判定能否达到根治性切除的标准或单纯行支气管肺动脉成形的可能；然后对下叶支气管、下肺静脉和基底干肺动脉尽可能地游离暴露，决定下肺叶是否达到再植入的条件，最后处理下肺静脉避免肺充血和水肿。可选择血管闭合器切断下肺静脉及肺直线切缝器处理发育不全肺裂或受侵及下肺组织，能大大缩短肺缺血的时间。首先吻合肺静脉，一般应用5-0 Prolene连续全层外翻缝合，尽量避免牵拉或钳夹损伤血管壁，避免形成血栓。肺静脉吻合后立即开放阻断钳，使左心房血液逆流灌注再植肺，缩短肺缺血时间，当肺动脉干流出无血栓颗粒的血液后，阻断肺动脉远端。用3-0可吸收微桥缝线行膜部连续、软骨部单纯间断缝合，缝合间距和边距约为1.5 mm-2.0 mm较好，尽量使两断端口径一致，远侧可修成斜面以增大面积便于调整口径大小不一的问题，严格将软骨环对软骨环、膜状部对膜状部，并将线结打在管腔外。微桥线对于减轻吻合口水肿程度、减轻刺激性咳嗽效果较佳^[2]^。文献报道采用肋间肌瓣或胸膜包盖吻合口是减轻吻合口瘘的好方法，我们认为由于肺动脉的侧支循环丰富，若吻合口处不存在明显水肿或血供较差的情况，不必常规行吻合口包埋，应用化学胶局部喷洒效果较好。用5-0 Prolene连续全层外翻吻合肺动脉，末针等待开放的上肺静脉血反流涌出时打结，完成再植手术的主要步骤。

### 2.3 安全有效的抗凝措施

预防肺动脉血栓形成和肺静脉栓塞是手术成功的关键。在进行再植肺叶切除前，先行试阻断肺动脉2次，每次5 min，间隔5 min，然后正式阻断或直接切除肺动脉，据动物实验所得经验，这样可以增加离体肺抗缺血-再灌注损伤的能力^[3]^。抗凝治疗的要点：必须给予肝素抗凝，在肺动脉阻断前可以直接向肺动脉注射肝素溶液，亦可从外周静脉注射肝素溶液。我们选择全身半肝素化，术中在吻合血管时，应用肝素溶液冲洗吻合口。术后从静脉注射肝素钙维持7 d以上，避免肺静脉栓塞造成的手术失败，7 d以后改用华法林口服较安全，大约低强度抗凝3个月。应用抗凝剂过程中及时监测并记录凝血酶原时间，及时调整抗凝药物的剂量，直至停止抗凝治疗。

### 2.4 尽可能减轻离体肺的缺血-再灌注损伤

自体肺再植离体肺的灌注和保存问题还没有成熟统一的标准和答案，有文献^[4]^报道短时间内大的实体器官以15 ℃-25 ℃保存最佳，但没有具体给定肺的保存温度。Michael等^[5]^在23 ℃溶液中进行肺动脉灌洗，获得了良好效果。Starnes等^[6]^应用4 ℃的改良Euro-collins液交替灌注离体肺的肺动脉和肺静脉，直到肺组织变成白色，通空气直到肺基本膨胀，以无创钳封闭支气管保存在4 ℃冷盐水中，获得了良好的临床效果；一些学者采用接近室温（18 ℃-23 ℃）肝素盐水灌洗和保存离体肺取得成功^[7, 8]^。总之使肺组织尽可能的保存在与其相近的生理环境中，减少肺组织缺血再灌注损伤是研究的最终目的。在同种异体肺再植中供肺的保护是肺再植成功的关键因素之一，低钾右旋糖酐（low-potassium dextran solution, LPD）液是专为肺保存设计的液体，是一种基于血液成分的细胞外液型保存液，低钾和右旋糖苷40是其关键成分。低钾可以保持内皮细胞结构和功能的完整性、减轻肺血管的收缩；右旋糖苷通过适当提高胶体渗透压，可减轻肺组织水肿和改善微循环^[9]^；同时，LPD溶液的电荷组成、渗透压，稳定了中性粒细胞膜，减少参与炎性反应的白细胞数量，并抑制其在以后再灌注时的活化反应。我们在检阅相关文献和汲取异体肺再植临床经验的基础上，采用低温LPD液顺、逆行交替灌注离体肺并保存在4 ℃肝素溶液中可防止微血栓形成聚积于血管系统中，本组3例患者围术期恢复良好。

### 2.5 控制术后感染，促进肺复张

再植肺支气管神经损伤、远端纤毛运动减弱及吻合口的机械屏障作用容易造成吻合口远端分泌物潴留，而并发肺不张和肺感染。术后应用有效抗生素控制感染，给予雾化吸入，保持胸腔闭式引流通畅，适当应用强的松、地塞米松类药物，鼓励患者努力咳嗽排痰；术后常规纤支镜检查，彻底吸痰，观察吻合口情况。这些是保证手术成功的关键。早期出现全肺不张或部分肺膨胀不全，其主要原因是吻合口水肿，早期积极行经纤维支气管镜吸痰，能有效减轻吻合口水肿，抑制肉芽组织增生，更有利于术后恢复，促使肺的复张。

总之，通过对3例患者的治疗，我们体会到自体肺叶再植不应仅限于心肺功能不全、不能耐受全肺切除术的上叶中心型肺癌患者，这是一种较为理想的肺癌根治术式；应根据医院实际情况尽可能地选择自体肺叶再植，最大限度地切除病变和保留健康肺组织，以提高患者的术后生存质量，但前提是必须满足在可完成根治性全肺切除的基础上进行；充分的围术期准备及管理、全面细致的术中探查、安全有效的抗凝措施和尽可能地减轻离体肺的缺血-再灌注损伤是肺叶再植成功的关键。
